# Supplementary material for: Toxic and Phenotypic Effects of AAV_Cre Used to Transduce Mesencephalic Dopaminergic Neurons
Source: Int J Mol Sci. 2022 Aug 21;23(16):9462. doi: 10.3390/ijms23169462 (PMC9408874; doi:10.3390/ijms23169462)
Supplement: Supplementary file 1 [file ijms-23-09462-s001.zip › ijms-1819719-supplementary.pdf]

Supplementary Materials

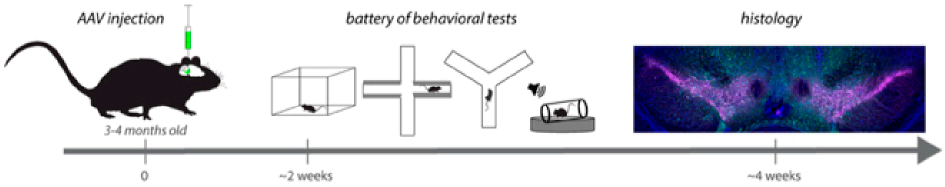

figure / experiment	strain	hemisphere	control	Cre	concentration [GC/mL]	exclusion
1	ErbB4 Cyt-1$^{fl/fl}$	bilateral	AAV1_syn_GFP n=8	AAV1_syn_Cre-GFP n=9	$3 \cdot 10^{13}$	n= 2 (misinjection) n=1 (dead)
2	ErbB4 Cyt-1$^{fl/fl}$	bilateral	AAV9_syn_GFP n=9	AAV9_syn_Cre + AAV9_syn_DIO_GFP n=9	$3 \cdot 10^{13}$	n= 2 (misinjection)
3	histological analysis of Figure 1 & Figure 2					
4	C57BL/6J	unilateral	AAV1_syn_GFP n=5	AAV1_syn_Cre-GFP n=6	$3 \cdot 10^{13}$	
5	C57BL/6J	bilateral	AAV9_syn_GFP n=11	AAV9_syn_Cre + AAV9_syn_DIO_GFP n=11	$3 \cdot 10^{12}$ (Cre: $1 \cdot 10^{10}$)	n= 3 (misinjection)

Figure S1. Study design and timeline of experiments. Wild-type C57BL/6J and ErbB4 Cyt-1$^{fl/fl}$ adult mice (3-4 month old) were injected uni-/bilateral with AAV (see table) into the VTA. Two weeks after injection mice were subjected to a series of behavioral analyses (open field, elevated plus maze, Y-maze and prepulse inhibition) and histological analyses were performed 4 weeks after injection. Different experiments (Figures 1,2,4,5) tested different AAVs and concentrations summarized in the table. n = number of animals tested.

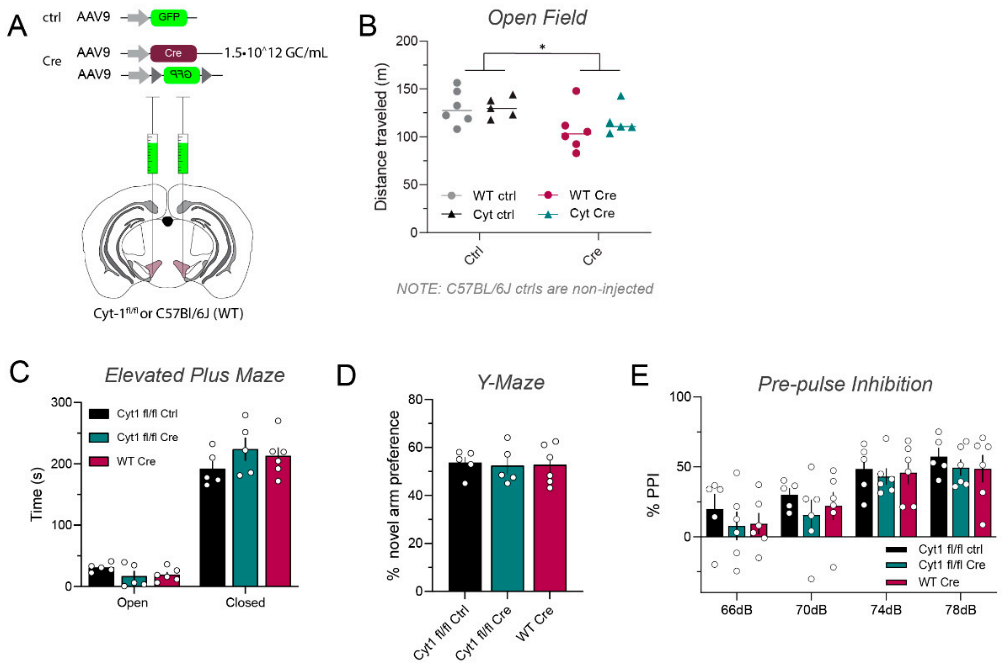

Figure S2. Mice with VTA injection of AAV9_Cre (10^{12} GC/mL) exhibit increased activity in the open field. (A) Scheme visualizing bilateral injection of AAV9_Cre (1.5 x 10^{12} GC/mL) mixed with AAV9_DIO_GFP (2.85 x10^{13} GC/mL; mixture final concentration 3 x 10^{13} GC/mL and hereafter denoted as Cre) and control AAV9_GFP (3.0 x10^{13} GC/mL) into the VTA of adult Cyt-$1^{fl/fl}$ and WT C57BL/6J mice. (B) Total distance traveled in the open field is significantly different between control and Cre injected mice, independent of genotype (n=5-6/group; two-way ANOVA, $F(1, 18)=0.4462$, $p=0.5126$, virus $p=0.0210$ & genotype = 0.5427). (C) Anxiety assessed in the elevated plus maze is unaffected in Cre- and control-injected mice (n=5-6/group, two-way ANOVA, $F(2, 26)=2.046$, $p=0.1495$ & genotype $p=0.7452$). (D) Working memory measured via novel arm preference in the Y-Maze remained intact across all groups (n=5-6/group, Ordinary one-way ANOVA, $F(2, 13)=0.03942$, $p=0.9615$). (E) Sensorimotor gating is largely unaffected (n=5-6/group, two-way ANOVA, $F(6, 56)=0.08610$, genotype $p=0.2586$).
